# Adaptive P300-Based Brain-Computer Interface for Attention Training: Protocol for a Randomized Controlled Trial

**DOI:** 10.2196/46135

**Published:** 2023-07-05

**Authors:** Sandra-Carina Noble, Eva Woods, Tomas Ward, John V Ringwood

**Affiliations:** 1 Department of Electronic Engineering Maynooth University Maynooth Ireland; 2 Department of Biology Maynooth University Maynooth Ireland; 3 Insight Science Foundation Ireland Research Centre for Data Analytics Dublin City University Dublin Ireland

**Keywords:** ADHD, attention, BCI, brain-computer interface, cognitive deficit, cognitive disease, cognitive training, dementia, EEG, electroencephalography, ERP, event-related potential, neurodegeneration, neurofeedback training, P300 speller, stroke

## Abstract

**Background:**

The number of people with cognitive deficits and diseases, such as stroke, dementia, or attention-deficit/hyperactivity disorder, is rising due to an aging, or in the case of attention-deficit/hyperactivity disorder, a growing population. Neurofeedback training using brain-computer interfaces is emerging as a means of easy-to-use and noninvasive cognitive training and rehabilitation. A novel application of neurofeedback training using a P300-based brain-computer interface has previously shown potential to improve attention in healthy adults.

**Objective:**

This study aims to accelerate attention training using iterative learning control to optimize the task difficulty in an adaptive P300 speller task. Furthermore, we hope to replicate the results of a previous study using a P300 speller for attention training, as a benchmark comparison. In addition, the effectiveness of personalizing the task difficulty during training will be compared to a nonpersonalized task difficulty adaptation.

**Methods:**

In this single-blind, parallel, 3-arm randomized controlled trial, 45 healthy adults will be recruited and randomly assigned to the experimental group or 1 of 2 control groups. This study involves a single training session, where participants receive neurofeedback training through a P300 speller task. During this training, the task’s difficulty is progressively increased, which makes it more difficult for the participants to maintain their performance. This encourages the participants to improve their focus. Task difficulty is either adapted based on the participants’ performance (in the experimental group and control group 1) or chosen randomly (in control group 2). Changes in brain patterns before and after training will be analyzed to study the effectiveness of the different approaches. Participants will complete a random dot motion task before and after the training so that any transfer effects of the training to other cognitive tasks can be evaluated. Questionnaires will be used to estimate the participants’ fatigue and compare the perceived workload of the training between groups.

**Results:**

This study has been approved by the Maynooth University Ethics Committee (BSRESC-2022-2474456) and is registered on ClinicalTrials.gov (NCT05576649). Participant recruitment and data collection began in October 2022, and we expect to publish the results in 2023.

**Conclusions:**

This study aims to accelerate attention training using iterative learning control in an adaptive P300 speller task, making it a more attractive training option for individuals with cognitive deficits due to its ease of use and speed. The successful replication of the results from the previous study, which used a P300 speller for attention training, would provide further evidence to support the effectiveness of this training tool.

**Trial Registration:**

ClinicalTrials.gov NCT05576649; https://clinicaltrials.gov/ct2/show/NCT05576649

**International Registered Report Identifier (IRRID):**

DERR1-10.2196/46135

## Introduction

### Background

Cognition includes many facets of complex processing functions, such as, and not limited to, attention, the acquirement of knowledge, memory, decision-making, and comprehension [1]. Cognitive deficits often present as symptoms in many underlying neurological disorders.

In the aging population, cognitive decline is common, however, can be accelerated by neurodegenerative diseases, such as dementia, or through brain trauma and stroke. Furthermore, the increased prevalence of cognitive deficits is apparent in every age demographic. As a result of the COVID-19 pandemic, many long-term neurological effects have been identified in patients who have experienced COVID-19 infection. In a study published by Hartung et al [2], it was identified that of a sample group of 969 patients, approximately 26% of patients experience mild cognitive impairment in the aftermath of the infection. Other cognitive diseases, such as attention-deficit/hyperactivity disorder (ADHD) and autism spectrum disorder, are common neurocognitive disorders that are identified in children. All these diseases feature a profound effect on the quality of life of the individual because of impairments in cognition [3-5].

The common treatments for these conditions are pharmacological interventions or therapeutic interventions. However, these disorders have no known cure, and rather, treatments are created to alleviate the symptoms of the disorder.

Pharmacological interventions, such as the use of psychostimulant medicines, have been rendered highly effective in treating symptoms of cognitive deficits. However, they have been criticized for the adverse effects which can occur due to their use [6]. Methylphenidate and lisdexamfetamine are 2 common psychostimulants used in the treatment of a variety of cognitive disorders, including ADHD. Both stimulants have shown to be efficacious in the treatment of the symptoms associated with the disorder but can result in insomnia, decreased appetite, headaches, and abdominal pain [7].

Cognitive disorders and their interventions, both pharmacological and nonpharmacological, can be highly costly, not only for the individual but also for health care systems globally [8,9]. This can cause great distress for individuals, as diagnosis and treatment for cognitive disorders can become inaccessible, as well as immense pressure on health care systems due to the increasing burden of these diseases. For these reasons, there is a clear need for low-cost and effective treatments for neurocognitive disorders.

### Neurofeedback Training

Electroencephalographic neurofeedback (EEG-NFB) is a type of brain-computer interface (BCI), which provides real-time feedback to the individual based on neural signals of interest as measured by electroencephalography (EEG), thus training the individual to self-regulate their brain activity [10]. EEG-NFB was introduced in the 1960s independently by Nowlis and Kamiya [11], and Sterman et al [12], and was commonly referred to as EEG biofeedback. Nowlis and Kamiya [11] presented data to confirm that patients could control their brain activity as a result of engaging with rewards using an EEG measuring device. Sterman et al [12] discovered through training cats that they too could modulate brain wave activity. As a result of these discoveries, a variety of treatments have been developed to alleviate symptoms of many cognitive disorders, including ADHD, schizophrenia, and many others [13-15].

Due to criticism relating to the efficacy of EEG-NFB in the 1970s, there was a decline in research on the use of EEG-NFB [16]. However, in recent years there has been a reemergence of the use of EEG-NFB globally in many research and clinical settings [10].

One example of a novel EEG-NFB is the adaptive P300 speller for attention training by Arvaneh et al [17]. The P300 speller was first introduced by Farwell and Donchin [18] in 1988 as a tool for patients who could not communicate, such as in the case of patients who have “locked-in” syndrome. In the speller, a matrix of symbols (eg, letters and numbers) is presented to the user. Each row and column in the matrix of symbols is highlighted or flashed in random order. When the user only focuses on the flashes of a target symbol and ignores the other flashes, the BCI can select this target symbol by identifying the row and column that elicited a P300 wave, thus allowing the user to spell words with their mind. In this process, each row and column are flashed several times, so that the signal-to-noise ratio (SNR) of the EEG signals can be increased by averaging. Usually, this number of flashes per row and column is fixed to allow the user to spell words sufficiently well in an acceptable amount of time. However, Arvaneh et al [17] progressively reduced the number of flashes in a training session based on the user’s performance to intentionally decrease the SNR. This makes it more difficult for the user to spell words correctly, thus encouraging them to improve their focus in order to maintain their performance. The researchers demonstrated that a single training session on healthy adults resulted in an increase in performance in a cognitive task, as well as an enhancement of event-related potentials (ERPs) [17].

This study intends to replicate the experiment by Arvaneh et al [17], comparing their adaptation of the number of flashes to a novel adaptation approach based on iterative learning control (ILC).

### Framework of ILC

ILC is a type of control engineering that was introduced by Ukiyama [19] in the late 1970s and Arimoto et al [20] in the 1980s. ILC is applied to repetitive processes, where the same tracking task is repeated several times. Examples of traditional application areas of ILC are the control of pick-and-place industrial robots or semiconductor manufacturing [21]. ILC exploits the repetitiveness of processes such as these by learning from past experiences, thus reducing the tracking error over time, as well as adapting to small changes in the processes. An iterative learning controller is able to learn from past experiences by taking the control input and error of previous iterations into account when determining the control input for the next iteration. The typical framework for ILC is

*u_k_*_+1_(∙)=f(*e_k_*_+1_(∙),…,*e_k_*_–_*_s_*(∙),*u_k_*(∙),…,*u_k_*_–_*_r_*(∙)), **(1)**

where *k* is the iteration index, *u* is the control input, and *e* is the tracking error. There are different ways to determine the function *f* in (1). The simplest ILC algorithm is Arimoto et al’s [20] algorithm, where the next control input is equal to the control input plus the scaled error of the previous iteration, that is,

*u_k_*_+1_(*t*)=u_k_(*t*)+*γe_k_*(*t*+1), **(2)**

where *t* is discrete time, and *γ* is a scalar controller parameter. ILC algorithms generally take the form

*u*_k+1_(*t*)=*u*_k_(*t*)+*Ke_k_*(*t*+1), **(3)**

where *K* is the so-called learning-gain operator, which is often based on a model of the system, such as the inverse-model ILC algorithm [22] or the Adjoint algorithm [23]. The algorithms described in (2) and (3) are so-called P-type ILC algorithms as they use the error from previous iterations. Algorithms that use the derivative of the error instead are known as D-type ILC [24].

While ILC was mostly used in industrial and manufacturing processes in its early days, it has found popularity in other application areas, such as stroke rehabilitation [23] or the control of exoskeletons [25] in more recent years.

Since rehabilitation and training, whether physical or cognitive, are inherently repetitive processes, where the task difficulty needs to change progressively, this application area lends itself nicely to the use of ILC.

### Objectives

The primary objective of this study is to accelerate P300-based attention training by progressively and optimally adjusting the task difficulty in a training session to each individual using ILC. The proposed iterative learning controller, which determines the task difficulty based on previous performance and task difficulty level, will be compared to the adaptation algorithm used in a previous study by Arvaneh et al [17]. Thus, we will be able to analyze the replicability of Arvaneh et al’s [17] study. A random task difficulty control group will also be used in this study to compare the training effects of personalized approaches with the training effects due to exposure to a range of nonpersonalized task difficulty levels.

While we expect the cognitive enhancements achieved during the training session to be similar among the groups, we expect the experimental group, where the task difficulty is determined by an iterative learning controller, to complete the training session more quickly than the other groups.

## Methods

### Study Design

In this single-blind, 3-arm randomized controlled trial with parallel-arm design, the effectiveness of different task difficulty adaptation approaches in a P300 speller task will be evaluated and compared. The study involves a single experimental session that lasts no longer than 2 hours (including setup). The experiments will be conducted in an electrically shielded, dark, and quiet room on the Maynooth University campus.

### Recruitment

The participants for this study will be recruited from the staff and student community of Maynooth University through an open enrollment process. Study participants will be alerted to the study through advertisement flyers distributed throughout the Maynooth University campus and through email advertisements sent to staff and students.

### Participants

The participants will be healthy individuals aged 18 years or older with no self-reported history of neurological or cognitive illness, and normal or corrected-to-normal vision. Interested participants who do not meet these criteria, have a negative reaction to the electroconductive gel, or who are illiterate will be excluded from the study.

Based on previous work using a P300-based BCI for attention training [17,26], a sample size of 45 participants (15 per group) was determined. Participant recruitment will continue until the sample size is reached. It is expected that around 50 participants will be recruited due to a 10% dropout rate.

### Ethical Considerations

This study was approved by the Maynooth University Ethics Committee (BSRESC-2022-2474456) and is registered on ClinicalTrials (NCT05576649). Interested participants who meet the eligibility criteria and give informed consent to study participation, allowing the recording of their performance in the training session and their brain signals, as well as the use of the collected data for this study and future research, will be invited to conduct a patch test to mitigate the risk of allergic reactions to the electroconductive gel. Participants will be advised that they have the freedom to withdraw from the experiment at their discretion at any time throughout the course of the experiment and will not be compensated for their participation in the study. The data from this study will be irreversibly anonymized immediately upon collection.

### Procedure

**Figure 1:**
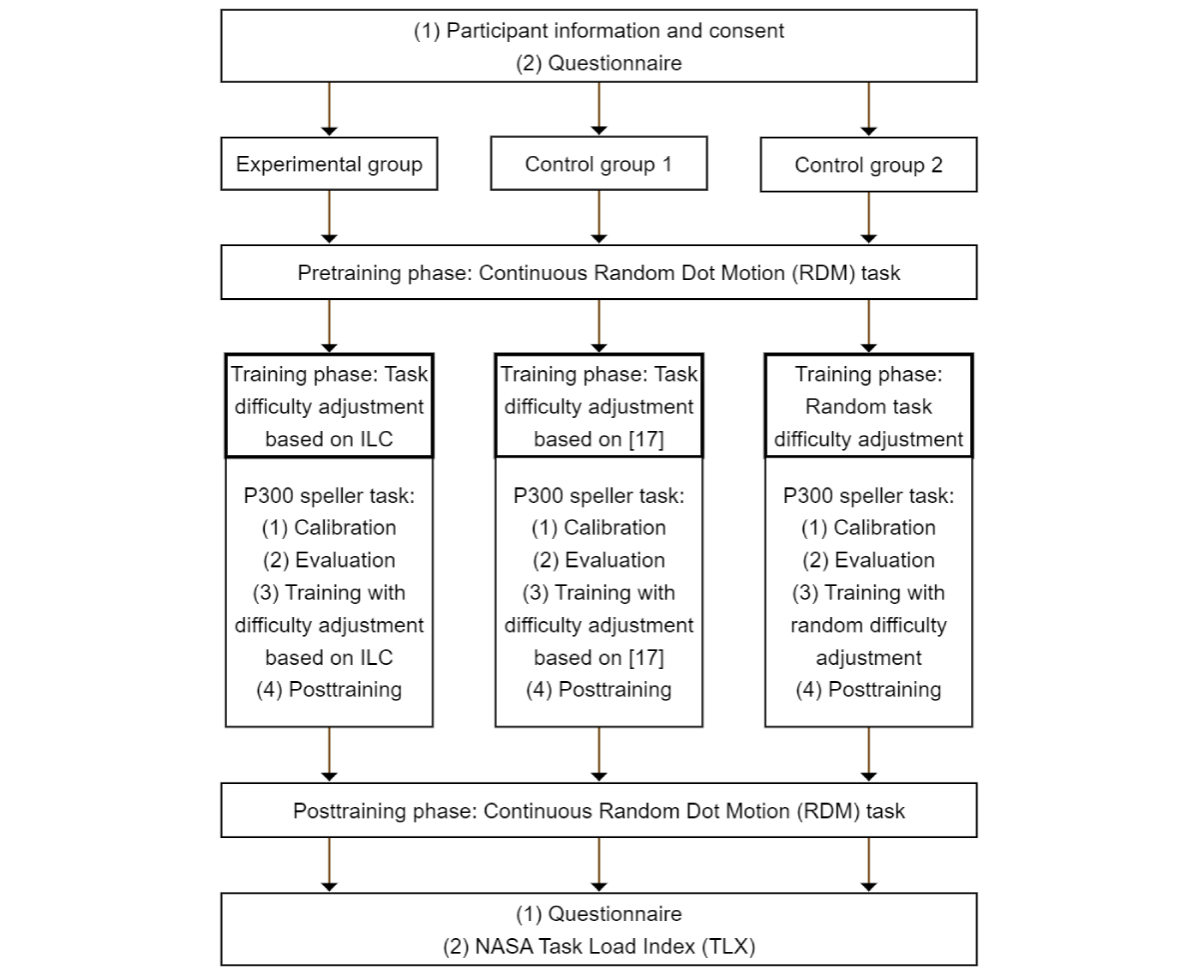
Study procedure based on Arvaneh et al [17]. ILC: iterative learning control.

The experimental procedure of this study is based on previous work by Arvaneh et al [17]. An overview of the procedure can be found in Figure 1.

Participants will be asked to fill in a questionnaire at the beginning and end of the experiment. They will then complete a continuous random dot motion (RDM) task, which will serve as a baseline for their cognitive abilities. Afterward, participants will receive attention training using a P300 speller task, where the task difficulty in each run of the speller is either chosen randomly (control group 2) or adjusted to the participant’s performance (control group 1 and experimental group). To analyze improvements in cognitive abilities, participants are asked to complete the RDM task again after the training.

### EEG Data Acquisition

Participants’ EEG data will be recorded during the P300 speller training phase of the experiment. EEG data will be collected using the battery-operated ANT Neuro eego rt [27] with a 32-channel waveguard cap [28]. The sintered silver–silver chloride electrodes are located in standard 10-20 positions. Impedances will be maintained below 5 kΩ during recording.

The EEG signals will be acquired in the OpenViBE software [29] at a sampling rate of 500 Hz. All channels will be band-pass filtered (1-20 Hz) and down-sampled by a factor of 4 before further processing.

Offline analysis of the EEG data will be carried out in MATLAB [30] using the EEGLAB toolbox [31]. Since this study is a replication of Arvaneh et al’s [17] study, the following offline analysis steps closely follow the steps described in the study. The continuous EEG signals will be rereferenced to Fz, band-pass filtered between 0.5 and 35 Hz and separated into baseline-corrected epochs of 150 milliseconds before the stimulus to 550 milliseconds post stimulus, where the 150 milliseconds prestimulus period is used as the baseline. Epochs with an amplitude of more than 75 µV or those with a voltage step of more than 150 µV within a 200-millisecond window will be excluded from analysis.

### Tasks and Stimuli

#### Questionnaire

Participants will be asked to complete the same 10-point Likert scale questionnaire as in Arvaneh et al [17]. It consists of the four questions: (1) How tired are you now? (2) How alert do you feel? (3) How bored do you feel? (4) Do your eyes feel tired?

This questionnaire will be completed at the beginning and end of the experiment so that responses before and after training can be compared.

In addition, the NASA Task Load Index will be used at the end of the experiment to assess participants’ perceived workload of the training [32].

#### RDM Task

The continuous RDM task is based on Arvaneh et al [17] and Kelly and O’Connell [33]. In this task, the participants will focus on moving dots on the screen, which transition from incoherent motion to coherent motion. During incoherent motion, all dots will continuously move in random directions, whereas in coherent motion, a significant fraction of the dots will move in one direction, either left or right. The participants will be asked to indicate the direction of the coherent motion by pressing the left or right arrow key once they are sure of the motion direction. In both the pre- and posttraining phases, the main task consists of 40 trials, where the coherence level, that is, the fraction of coherently moving dots, and the motion direction vary in each trial.

An average of 118 dots, with a size of 6×6 pixels each, are displayed in a circular aperture of 5° at a viewing distance of 70 cm, resulting in a dot density of approximately 10.8%. Each dot is black against a grey background. The dots are moving at a speed of 3.33°/second. The incoherent motion lasts for either 3.1, 4.2, or 5.7 seconds, and the coherent motion always lasts for 1.9 seconds. The coherence level is either 19% or 25%.

To allow participants to understand the task, a practice session will be carried out before the pretraining phase. The practice session consists of 3 blocks of 6 trials each. In the first block, the coherence levels are either 60% or 80%, reducing to 30% or 40% in the second block, and finally reaching 20% or 25% in the last block of the practice session. All other parameters of the task are the same as above. During the practice session, participants will receive verbal feedback on hits, misses, and false alarms.

The code for this task was developed in-house using PsychoPy [34] and the stimuli are presented on a 52.7-cm-wide LED monitor with a refresh rate of 60 Hz and a resolution of 1920×1080 pixels.

#### P300 Speller Task

##### Overview

In this study, the P300 speller implementation in OpenViBE [29] is used. This P300 speller uses the xDAWN algorithm [35] to train a spatial filter that enhances the SNR of the P300 ERPs. This reduces the 32 EEG channels to 3 components, which are weighted combinations of all channels. A linear discriminant analysis classifier is used to distinguish target and nontarget trials. It should be noted that the row and column with the highest probability of belonging to the target class are chosen, even if that is below 50%. Both the spatial filter and classifier are trained for each participant using calibration data.

**Figure 2:**
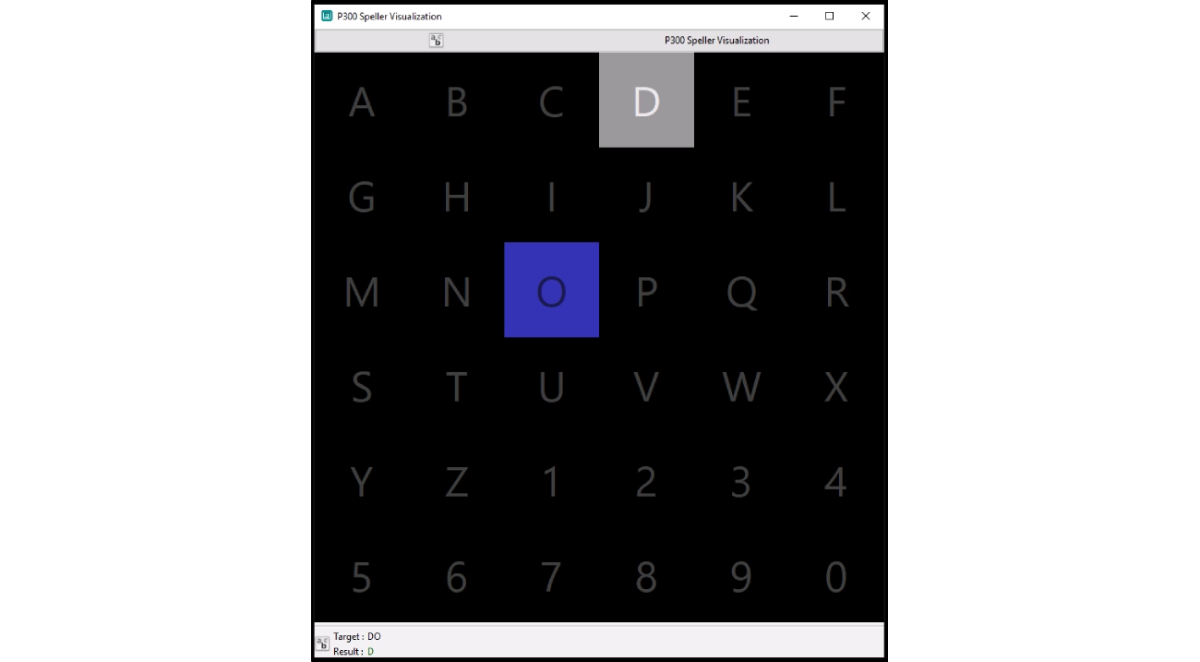
OpenViBE [29] implementation of the P300 speller. The next target letter in the word “dog,” “o,” is currently highlighted in blue. The previous letter, “d,” was correctly identified by the speller and is therefore highlighted in grey. The target symbols are displayed below the grid of letters and numbers (“Target:”), and the previously identified symbols can be seen below the target (“Result:”).

Figure 2 shows the 6 by 6 grid of letters and numbers that is displayed on the screen. The word to be copy-spelled and the identified symbols can be seen below the grid. If the identified symbol is the same as the target symbol, it will be displayed in green. If the identified symbol is in the same row or column as the target symbol, it will be displayed in orange and if it is wrong, it will be displayed in red.

Outside of the training phase, 12 flashes per row and column are used. Each flash lasts 55 milliseconds with an interflash interval of 117 milliseconds. Each target symbol is highlighted in blue for 6 seconds before the flashing begins.

In each run after the calibration phase, the spelling accuracy, that is, the fraction of symbols that were identified correctly, is calculated for all numbers of flashes between 1 and the actual number of flashes used.

##### Calibration Phase

The calibration phase is used to collect training data for the spatial filter and classifier. The participants will complete 2 runs, where the words “the” and “quick” are copy-spelled without feedback. The recorded EEG signals from both runs are concatenated and then used to first train the xDAWN spatial filter before the linear discriminant analysis classifier is trained. Both target and nontarget trials are separated into epochs ranging from stimulus onset to 600 milliseconds poststimulus. The spatial filter is trained using the continuous EEG signals as well as the target epochs, whereas the classifier uses target and nontarget epochs for training.

##### Evaluation Phase

Once the spatial filter and classifier are trained, the participants will copy-spell the word “dog.” If at least 2 of the 3 letters are correctly identified by the P300 speller, the participant will move on to the training phase. Otherwise, the spatial filter and classifier will be retrained with the added EEG signals from the latest run. They will then copy-spell the word “fox.” If only 1 of the 3 letters is identified correctly, the participant will be removed from the study.

##### Training Phase

During the training phase, all participants will copy-spell the word “beautiful” for 5 runs. Ten flashes per row and column are used for the first run of the training phase, for all groups. The number of flashes in all subsequent runs is adapted randomly (control group 2) or based on the participants’ performance in the previous run (control group 1 and experimental group). The participants in all groups will receive feedback but will not be told why the number of flashes varies in each run.

The details of the task difficulty adaptation for each group are described below:

*Control group 1*: The adaptation algorithm in control group 1 is a replication of the work described in [17]. The number of flashes in each run, *N_i_*, is the average of the minimum number of flashes needed to reach more than 66% spelling accuracy in the previous run, referred to as *N*_(*i*–1)66_, and the actual number of flashes used in the previous run, *N*_*i*–1_, that is,



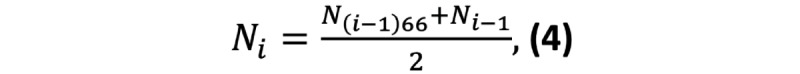



where *N_i_* is rounded to the nearest integer. If the spelling accuracy in the previous run was below 66%, then the number of flashes in the next run is increased by 1, that is,

*N_i_*=*N_i_*_–1_ + 1. **(5)**

*Control group 2*: The task difficulty adaptation of control group 2 is not based on the participants’ performance. Instead, the number of flashes in each run is chosen at random between 1 and 10 flashes. Choosing the number of flashes in this way will expose the participants in control group 2 to varying task difficulties in an unpredictable way. This allows the authors to compare the personalized adaptation approaches of the other 2 groups to a nonadaptive difficulty adjustment approach under similar conditions. Using a constant task difficulty in this group would potentially lead to differences due to an easier average task difficulty [36] and reducing the number of flashes by the same amount each run would introduce predictability, which the other groups do not have.

*Experimental group*: In the experimental group, a P-type ILC controller is used to adapt the number of flashes in the next run, *N_i_*, based on the number of flashes and error in the previous run, *N_i_*_–1_ and *e_i_*_–1_=1-*J*_1( *i*–1)_ (where *J*_1_ is the spelling accuracy). The update law of the controller is

*N_i_*=*N_i_*_–1_+ϵ*f*(*e_i_*_–1_), **(6)**

where ε is a controller parameter and

*f*(*e*_i–1_)=2*e_i_*_–1_–1. **(7)**

**Figure 3:**
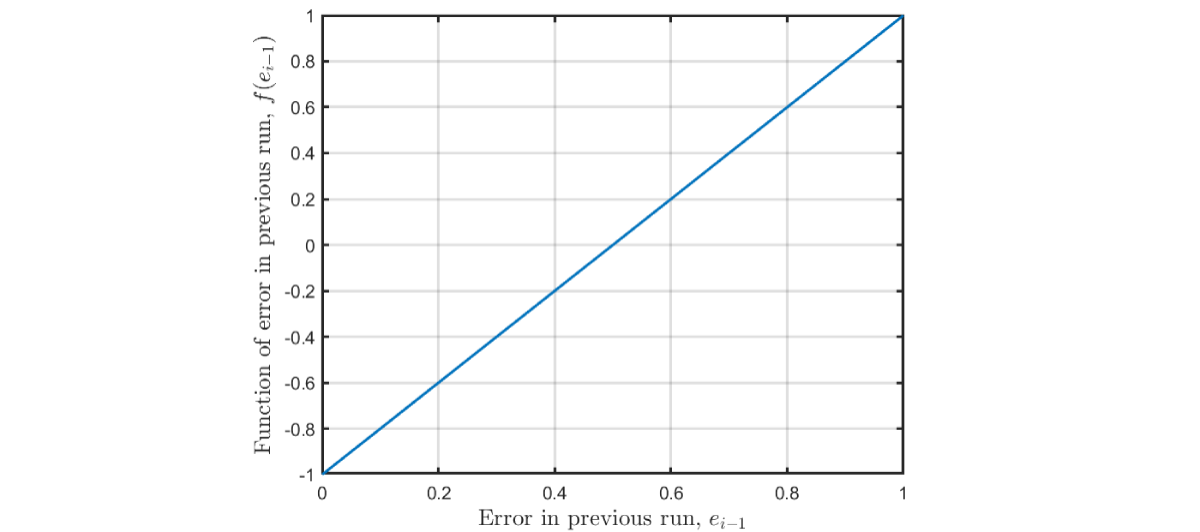
The function of the error in the previous run, f(e_i–1_), used in the P-type ILC update law, plotted against the error in the previous run, e_i–1_.

Figure 3 shows *f*(*e_i_*_–1_) against *e_i_*_–1_. As can be seen, if only half of all symbols in the previous run were identified correctly, that is, 50% error, *f*(*e_i_*_–1_) will be 0, which means that the number of flashes in the next run will remain the same as that in the previous run. *f*(*e_i_*_–1_) will be –1, if all symbols were identified correctly, and1 if no symbols were identified correctly. This means that the controller parameter *ϵ* is the maximum update step of the number of flashes from one run to the next. ε was tuned in simulation and pilot experiments to the following:



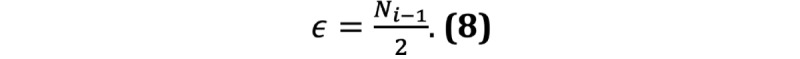



The update law described in (6), (7), and (8) can therefore be simplified to

*N_i_*=*N_i_*_–1_(*e_i_*_–1_+0.5). **(9)**

The new number of flashes, *N_i_*, is then rounded up to the next highest integer.

##### Posttraining Phase

After the training phase, which differs for each group and individual, the participants in all groups will copy-spell the word “dance” with 12 flashes per row and column. This is done to compare any posttraining differences between the groups.

### Data Analysis

The offline data analysis steps closely follow the steps described by Arvaneh et al [17] to allow for comparability of results.

#### Questionnaire

The answers to Arvaneh et al’s [17] questionnaire will be analyzed for between-group and within-group differences, using statistical tests, such as repeated measures ANOVA and paired *t* tests.

Similarly, between-group differences in responses to the NASA Task Load Index questionnaire [32] will be analyzed, for example, using a 1-way ANOVA. The raw score of each question will be used for the data analysis.

#### RDM Task

The change in performance in the RDM task between pre- and posttraining phases will be analyzed in terms of accuracy and response time. The accuracy is the number of correct trials over the total number of trials, and the response time is the elapsed time between onset of the coherent motion until the correct button is pressed before the end of the coherent motion. The response time of incorrect trials will not be considered. The mean response time will be calculated for each participant individually. Statistical tests, such as ANOVA and paired *t* tests, will be conducted to analyze between-group and within-group differences for both the accuracies and mean response times.

#### P300 Speller Task

##### Spelling Accuracy

The between-group differences in performance in the P300 speller in terms of spelling accuracy will be analyzed for all participants. Only the evaluation run, the first run of the training phase, and the posttraining run will be considered, as these 3 runs use the same number of flashes per row and column for all participants. Training runs 2-5 are different for each individual as the number of flashes is either determined by their previous performance (control group 1 and experimental group) or chosen randomly (control group 2). This means that the performance during these runs cannot be compared directly.

##### Length of Training

Since 1 of the objectives of this study is to accelerate attention training, the length of the training will be compared in terms of the total number of flashes used in runs 2-5 of the training phase. A statistical test, such as the 1-way ANOVA, will be carried out to compare between-group differences.

##### ERP Components

The changes in the ERP components throughout the training will be analyzed. For this purpose, epochs from 150 milliseconds to 550 milliseconds poststimulus onset will be extracted from the EEG data for both target and nontarget trials. Subsequently, the total power across the same centroparietal electrodes as used in [17] (ie, C3, Cz, C4, P3, Pz, and P4) will be calculated for each epoch.

The average total power for all epochs of the calibration and evaluation phases, the training phase, and the after-training phase, respectively, will be calculated. A training-to-calibration ratio of the average power for both target and nontarget trials will be obtained by dividing the average total power during the training phase by the average total power during the calibration and evaluation phases. The same will be done with the average total power during the after-training phase to obtain an after-training-to-calibration ratio.

A ratio of greater than unity indicates an increase in average total power, whereas a ratio of less than unity indicates a decrease in average total power compared to the calibration and evaluation phase. For target trials, an increase in power means that the participant improved their attention, and a decrease in power of nontarget trials can be interpreted as the participant being less distracted by nontarget flashes [17].

Appropriate statistical tests, such as ANOVA, will be conducted to analyze within-group and between-group differences in the aforementioned ratios.

##### Time-Frequency Analysis

Similarly, to the training-to-calibration and after-training-to-calibration ratios of the average total power, these ratios will be calculated for the power of the alpha rhythm (7-12 Hz) of nontarget trials that are not presented immediately after a target trial. The alpha power will be calculated for the same centroparietal electrodes as before, for a period of 150 milliseconds poststimulus.

The training-to-calibration alpha ratio is the average alpha power during the training phase divided by the average alpha power during the calibration and evaluation stages, and the after-training-to-calibration alpha ratio is the average alpha power during the after-training phase divided by the average alpha power during the calibration and evaluation stages. A ratio of less than unity means that a participant is less distracted by nontarget stimuli compared to the calibration and evaluation phases [17].

Between-group differences of the training-to-calibration and after-training-to-calibration alpha ratios will be analyzed using statistical tests, such as 1-way ANOVA.

##### Correlation Between EEG and RDM Performance

The potential correlation between the brain activity during the P300 speller phase of the training session and the performance in the RDM task will be investigated with tests, such as the Pearson correlation coefficient or the nonparametric Spearman correlation test. The training-to-calibration and after-training-to-calibration ratios of total power and alpha power will be used as performance metrics during the P300 speller task. Similar ratios will be obtained for the response times and accuracies of the RDM task by dividing the posttraining phase response times and accuracies by the pretraining phase response times and accuracies, respectively.

## Results

This study was approved by the Maynooth University Ethics Committee (BSRESC-2022-2474456) and is registered on ClinicalTrials.gov (NCT05576649). Participant recruitment and data collection began in October 2022. We expect to publish the results in 2023. This study is replicating the experimental protocol of Arvaneh et al [17], comparing their task difficulty adaptation approach to a novel approach using an iterative learning controller, as well as random task difficulty levels in each run.

Arvaneh et al [17] showed a significant enhancement of ERP components and performance in the RDM task after the P300 speller training, suggesting that participants’ attention improved. We expect such an improvement for all groups in this study, with changes in the length of the training between groups due to the different adaptation approaches.

## Discussion

### Overview

This study aims to evaluate the use of an adaptive P300 speller as a neurofeedback training tool for attention training. We aim to accelerate the training process using ILC and compare personalized and nonpersonalized task difficulty adaptation approaches. By replicating the experimental protocol of Arvaneh et al [17], who demonstrated the adaptive P300 speller’s potential as an easy-to-use and effective neurofeedback training tool, we seek to gather further evidence supporting this application for attention training.

The findings of this study will contribute to the understanding of how different adaptation approaches, both personalized and nonpersonalized, can impact the efficacy of neurofeedback training tools. These insights can guide the design of future BCI-based cognitive training and rehabilitation interventions. Accelerating the training with the P300 speller, without affecting its effectiveness or the users’ frustration levels, will make it a more attractive training option for people with cognitive deficits.

### Limitations

There are limitations to this protocol. The participants will only receive a single session of the neurofeedback training and therefore the long-term impacts of the neurofeedback training will not be obtained. Due to the short length of the training session, participants may not experience significant cognitive enhancements. The participants for this study will be healthy, and most likely young (due to recruitment in the university community) adults. It can be assumed that these individuals possess close to maximal cognitive abilities, which further limits the significance of the cognitive enhancements of the training.

### Conclusions

Due to an aging and growing population, the number of people with cognitive deficits and illnesses is rising. It is therefore important to develop cognitive training and rehabilitation tools that are affordable, easy to use, and effective. The adaptive P300 speller that will be evaluated in this study might have the potential to be an efficient training tool. Positive findings of this study may demonstrate that the adaptive P300 speller, and more broadly BCIs, can be effective tools in cognitive training and rehabilitation.

## Abbreviations

ADHD: attention-deficit/hyperactivity disorder
BCI: brain-computer interface
EEG: electroencephalography
EEG-NFB: electroencephalographic neurofeedback
ERP: event-related potential
ILC: iterative learning control
RDM: random dot motion
SNR: signal-to-noise ratio

## Data Availability

The data sets that will be generated during this study will be available in the Zenodo repository once work on this study has been completed.
